# On Cooperative Behavior in Distributed Teams: The Influence of Organizational Design, Media Richness, Social Interaction, and Interaction Adaptation

**DOI:** 10.3389/fpsyg.2016.00692

**Published:** 2016-05-12

**Authors:** Dorthe D. Håkonsson, Børge Obel, Jacob K. Eskildsen, Richard M. Burton

**Affiliations:** ^1^Department of Business Development and Technology, Aarhus UniversityHerning, Denmark; ^2^Department of Management, Interdisciplinary Center for Organizational Architecture, Aarhus UniversityAarhus, Denmark; ^3^Department of Culture and Society, Interacting Minds Centre, Aarhus UniversityAarhus, Denmark; ^4^Department of Management, Aarhus UniversityAarhus, Denmark; ^5^Fuqua School of Business, Duke UniversityDurham, NC, USA

**Keywords:** distributed teams, information sharing, opportunism, cooperation, media richness, organizational design, social interaction, interaction adaptation

## Abstract

Self-interest vs. cooperation is a fundamental dilemma in animal behavior as well as in human and organizational behavior. In organizations, how to get people to cooperate despite or in conjunction with their self-interest is fundamental to the achievement of a common goal. While both organizational designs and social interactions have been found to further cooperation in organizations, some of the literature has received contradictory support, just as very little research, if any, has examined their joint effects in distributed organizations, where communication is usually achieved via different communication media. This paper reviews the extant literature and offers a set of hypotheses to integrate current theories and explanations. Further, it discusses how future research should examine the joint effects of media, incentives, and social interactions.

## Introduction

Self-interest, defined as “regard for one’s own interest or advantage, especially with disregard for others^1^,” vs. cooperation, defined as “an act or instance of working or acting together for a common purpose or benefit,” is a classic dilemma.

In organizations, how to get people to cooperate despite or in conjunction with their self-interest is fundamental to the achievement of a common goal. The gains from cooperation are related to the ability to achieve a goal beyond the reach of the individual. The long-term benefits may be entry to the group or team. Yet, the returns may not be directly related to personal gains. The gains from self-interested behavior are immediate returns to oneself, whereas the longer-term consequences may be exclusion from the group or team.

Organization design theory contains a number of studies that examine how to best control self-interest and obtain collaboration via different designs and incentives (Burton and Obel, 1980, 1988; Williamson, 1985). It is less clear whether such design and incentive solutions will have a similar effect when interactions occur via media of different “degrees of richness” (Daft and Lengel, 1986). This seems important, however, as cooperation in today’s organizations is often achieved via different media, some of which (e.g., TelePresence or Skype) are “richer” and thereby better able to convey subtle meanings than others (e.g., e-mail or chat messages).

While the media richness approach offers concrete advice on which media to choose for effective cooperation (Daft and Lengel, 1986), results regarding the effect of media on cooperation remain contradictory. Further, whether the presence or absence of media influences people toward *self-interested behavior* remains an underexplored topic. One of the major criticisms of the media richness theory has been that it is too technologically deterministic (Markus, 1994; El-Shinnawy and Markus, 1997). Instead, it has been argued (Barley, 1988; Leonardi and Barley, 2008, 2010) that media can bring social structures that can both enable and constrain behavior. Social interactions (i.e., the processes where people act and react to others) have been shown to lead to the development of trust (Jarvenpaa and Leidner, 1998; Jarvenpaa et al., 2004; Hill et al., 2009), and cooperation, which, it is sometimes argued (e.g., Wilson et al., 2006), is a behavioral outcome of trust. However, empirical studies have not shown a consistent, positive effect of social interactions on people’s tendencies to behave cooperatively (e.g., Hancock et al., 2010). Theories of interpersonal adaptation hold interesting insights that may improve our understanding of the role of social interactions in communications. Yet these theories have not yet been integrated into the literature on distributed teams, just as they have not been examined across different media.

To shed light on the effect of media on cooperative vs. self-interested behavior, both organizational design theory and social interaction theory, as well as theories of interaction adaptation, hold relevant insights. However, the insights from these theories have not been related to each other in an integrated fashion. Toward this purpose, the paper reviews extant literature related to organizational design theory, media richness theory, social interaction theory, and interaction adaptation, and suggests a set of hypotheses based on current understanding. As the review will show, current understanding derived from these theories holds important, but not directly comparable insights, and some may even seem contradictory. Nevertheless, all of the different theories have empirical support. To integrate current understanding, and reconcile previously assumingly contradictory findings, the paper suggests a model that proposes an understanding of the joint effects of design and social interaction theories on cooperative vs. self-interested behavior in distributed teams (across different communication media). The model is intended to serve as a first step toward the development of testable hypotheses, and subsequent empirical analyses.

The paper proceeds as follows: First, we review the four theoretical perspectives: theories of organizational design; theories of media richness; theories of social interaction, and theories of interaction adaptation. Second, from our review, we develop hypotheses about how organizational incentives; media richness; social interactions, and interaction adaptation influences employees’ tendencies to behave cooperatively or out of self-interest. Finally, we develop our model to suggest an integration of current literature, and propose a set of interactional hypotheses, based on the model. The paper concludes with a discussion of our findings for future theory and empirical research.

## Theory and Hypotheses

### Organizational Design Theory and Cooperation vs. Self-interested Behavior

Parsons (1960:41), as quoted in Scott (2013), posited: “The development of organizations is the principal mechanism by which it is possible to get things done and to achieve goals beyond the reach of the individual.” Through cooperation and coordination, organizations allow individuals to achieve common and individual goals that they cannot achieve individually. Yet common goals may conflict with an individual’s self-interests.

Within organization theory, self-interested opportunism is ever present. Williamson (1985, p. 26) defined opportunism as “self-interest seeking with guile,” which to Williamson (1985, p. 47) includes lying, stealing, and cheating. More specifically, with respect to information, the individual can manipulate incomplete or distort the sharing of information, especially by modifying information to mislead, distort, disguise, obfuscate, or otherwise confuse partners in an exchange. Williamson’s (1985) notion of opportunism therefore does not relate to those situations where people, for example, withhold or distort information without realizing the potential benefits of doing so; rather, it relates to voluntary self-interested behavior, or cheating.

Not everybody behaves opportunistically; only some people do, and they only do so sometimes. However, since some people are prone to opportunism, it becomes important to design safeguards to protect oneself, or the organization, from opportunistic behavior. Traditionally, such safeguards are achieved via hierarchical managerial control (e.g., hierarchical governance; Williamson, 1985).

Following Williamson (1975), the advantage of hierarchy is that employees are directly responsible, or controlled, by a so-called third party. This third party, or manager, has what Williamson refers to as “managerial fiat,” meaning that (s)he has the right to resolve any conflict that emerges between employees. An organizational hierarchy can solve many problems, as employees who report to the same manager have less incentive to seek advantage over each other. Further, if disagreements should arise between employees, the third party has the authority to solve them. Yet hierarchy itself is subject to opportunism, as the manager is limited in his or her capacity to know all the relevant information and make the required decisions. Further, middle-level manages may have incentives that are not be aligned with the corporate profits (Burton and Obel, 1988).

Williamson’s (1975) line of argument led to a number of studies (e.g., Armour and Teece, 1978; Burton and Obel, 1980; Williamson and Ouchi, 1980; Williamson, 1985) demonstrating the efficiency of the M-form (multidivisional) over the U-form (unitary, or functional form) in handling strategic and operational planning.

The U-form and M-form are two alternative forms for organizing an organization. The U-form organizes by functions (e.g., marketing and manufacturing), and each function has its own middle manager. In the U-form the headquarters must coordinate the activities of the functions (e.g., manufacturing and marketing should align production and sales quantities). Here, the headquarters may well have less than complete or accurate information about the production or sales possibilities; in short, it relies on the manufacturing and sales functions to supply this information. Depending upon the incentive system or how the manufacturing and sales managers are rewarded, it may be advantageous for the manager to mislead the headquarters with less than truthful information; that is, the manager is engaging in opportunism.

In the M-form, manufacturing and sales are incorporated into the same managerial unit for coordinating manufacturing and sales. That is, the M-form organizes by products or markets (e.g., shoes and bags, or Europe and America). In the M-form, the headquarters has a different task of allocating resources; for example, operating monies or capital investments among the product or country units. Here, too, the M-form unit manager might be opportunistic by misleading the headquarters on the economic returns on the operations or investments. That is, the headquarters has incomplete information on what is possible. In short, opportunism is possible for both the U-form and the M-form due to incomplete information and incentives that encourage opportunism.

In a laboratory study, Burton and Obel (1988) investigated whether managers would be opportunistic in the M-form and U-form structures under corporate profit and divisional profit incentives. First, they found that managers were quite aware that opportunism was possible, i.e., they could influence their rewards by distorting the information; second, the managers knew what to do to take advantage of the situation, i.e., how to distort the information to their own benefit; and thirdly, some managers would behave opportunistically, some did not, and some were altruistic. However, there were differences between the M-form and U-form and between the corporate and divisional profits incentives.

Investigating M-form and U-form organizations under incentives based on corporate profit and divisional profits, Burton and Obel (1988) found that corporate profit incentive systems for cooperation led to less opportunism than the divisional profit incentive system. This was as expected. Based on that, we hypothesize:

Hypothesis 1a: An incentive system based upon corporate profit induces lower opportunism than an incentive system based upon divisional profit.

Burton and Obel (1988) further found that there was greater opportunism under the U-form organization. Not only was there greater opportunism, the negative effect on corporate profits was greater under the U-form. That is, opportunism for coordination in the U-form is more damaging than opportunism for allocation in the M-form. We therefore hypothesize:

Hypothesis 1b: When opportunism is present it will have a greater negative effect on corporate profit in the U-form than in the M-form.

They further tested different incentives schemes and showed how the organizational goal of subunit profit maximization created subunit incentives, which were not compatible with the overall corporate profit goals. Indeed, individuals sometimes misrepresented information to their own benefit, and at the potential expense of others (Burton and Obel, 1988). Hence, alternative organizational incentives induced differing degrees of information misrepresentations with varying impact on overall organizational performance (Burton and Obel, 1988).

The studies reviewed above constitute state-of-the-art thinking within organizational design theory on how organizational design and incentives may lead to opportunism but also may serve to reduce opportunism and increase cooperation. Nevertheless, organizations’ contexts have changed considerably since these theories were conceived. In today’s organizations, many, if not all organizational interactions take place using different media with colleagues who are not co-located, e.g., multinational corporations. Some studies have discussed the potential effects of virtuality on design solutions (Jensen et al., 2009, 2010). Surprisingly little, however, is known about whether design solutions and incentives also hold when organizational members are distributed and have to coordinate their actions via different media. Therefore it remains unclear as to whether design incentives will have the anticipated effect on cooperative vs. opportunistic behaviors in organizations in these situations.

### Media Richness Theory and Cooperative vs. Self-interested Behavior

Media richness theory was originally introduced by Daft and Lengel (1986), who proposed that communication media, because they differ in their ability to provide cues, have different capacities to reduce problems related to equivocality [i.e., what Daft and Lengel (1986) referred to as problems that were characterized by uncertainty but with the twist that they relate to ambiguous situations]. Following Daft and Lengel (1986), face-to-face is the richest medium because it provides immediate feedback and multiple cues via body language and tone of voice, and because it enables communicators to express themselves in natural language. Again following Daft and Lengel (1986, p. 560), rich media “facilitate equivocality reduction by enabling managers to overcome different frames of reference and by providing the capacity to process complex, subjective messages.” Media of high richness, such as face-to-face meetings, were therefore recommendable over written exchanges in solving what Daft and Lengel (1986) referred to as equivocal situations. In that sense, equivocal (or ambiguous) situations are different from uncertain situations. In uncertain situations, you know what it is that you need to know more about (e.g., you know whom your customers are and what products they want, but you don’t know how much they are likely to buy). With uncertainty, you can collect information to reduce your uncertainty. In equivocal situations, gathering more (factual) information may not help you solve the task, because the actual problem you face is that you don’t know what the problem is [see Burton et al. (2015) for a similar discussion]. Daft and Lengel’s (1986) theory was proposed as a theory to describe and evaluate the appropriateness of the usage of different media in solving different types of organizational tasks.

Since the theory’s conception, the effects of different media on cooperation have been widely studied by psychologists, organizational theorists, and communication scholars, providing numerous arguments in support of the idea that media of high richness enable people to work together more effectively, particularly on ambiguous tasks.

For instance, studies have shown that technological media of low richness reduce both the verbal and non-verbal cues that otherwise help the flow of conversation, facilitate turn-taking, and help convey subtle meaning (Straus and McGrath, 1994). Similarly, a lack of visual channels has been found to make coordination and cooperation more difficult in group decision-making tasks (Baltes et al., 2002), just as the inability to respond in real time (synchronously) has been found to lead to communication difficulties (Dennis et al., 2008). Relatedly, findings in the negotiation literature show that negotiators are less contentious and more cooperative when they can see their counterpart (Turnbull et al., 1976). A lack of cues has also been shown to impede relationship development and subsequent trust development (Jarvenpaa and Leidner, 1998; Jarvenpaa et al., 2004; Hill et al., 2009), lead to higher levels of conflict (Hinds and Bailey, 2003; Hinds and Mortensen, 2005), reduced cooperation (Naquin et al., 2008), and increased opportunistic defections (Bos et al., 2002; Rockmann and Northcraft, 2008).

Clearly, communication technologies have changed considerably since the original conception of the theory. Video and online conferencing have communication attributes that were not conceivable in the eighties. Whether new communication media may replace, or at least be comparable to face-to-face in their influence on cooperative vs. opportunistic behavior, is not clear. Similarly, while the above studies have examined the effects of media richness on cooperation, only a few studies have examined whether media richness has a similar, but opposite, effect on opportunistic behavior. Nevertheless, we find it reasonable to argue that:

Hypothesis 2: Employees who interact via media of high richness will behave less opportunistically than employees who interact via media of low richness.^2^

Despite the evidence that media richness matters, there are also a number of works that have failed to demonstrate a positive effect of media richness on cooperation.

For instance, in a laboratory study, Watson et al. (1988) failed to find support for their hypothesis that groups using group-decision support systems, designed to simulate decision-making when conflicting personal preferences were involved, would exhibit more consensus, greater equality of participation, and more confidence in their decisions.

### Theories of Social Interaction and Cooperative vs. Self-interested Behavior

A major criticism of the media richness theory has been that it is too technologically deterministic. As argued by DeSanctis and Poole (1994, p. 125): “advanced technologies bring social structures which enable and constrain interaction to the workplace.”

Consistent with this view, a number of field studies have indicated that the process by which individuals in an organization adopt (Fulk, 1993), interpret (Barley, 1988), and use (Poole and DeSanctis, 1990; Orlikowski and Yates, 1994) new technologies cannot be explained purely by the technological properties of the different communication media. Pre-existing social structures (Barley, 1986) as well as individual ways of thinking (Barley, 1988) can play significant roles in the use of communication media. Therefore, advanced information technologies can bring social structures, which can both enable and constrain information exchanges. While attempts have been made to integrate the media richness theory with social interaction theory (Swaab et al., 2012, social interaction theories generally hold that the social structures have preponderance over the effect of media). For example, Fulk (1993) found evidence that group members’ use of electronic mail was influenced by group members’ shared, identifiable patterns of meaning concerning media. Similarly, Schmitz and Fulk (1991) also found that social influences affected individuals’ use of new information technology.

Also supportive of this line of thinking, Walther (1994) found that the negative effects of computer mediated interactions over face-to-face interactions diminished over time. Following Walther (1992, 1994), people strive to develop positive and meaningful relationships. Even if computer-mediated technologies do not transmit social cues, which are important to the development of positive and meaningful relationships, at the same speed as face-to-face interactions, over time, users can adapt to their limitations and effectively develop interpersonal relations. Consistent with this model, decision makers who rely predominantly on text-based communication channels can be equally as successful as face-to-face teams if they take the time to establish a positive interpersonal connection (McGinn and Keros, 2002; Wilson et al., 2006).

Relatedly, Mortensen and Hinds (2001) found that geographically distributed teams who shared a strong identity experienced less conflict than distributed teams that did not share a strong identity. Hinds and Mortensen (2005) examined task and interpersonal conflict in both distributed and collocated teams. They found that distributed teams reported more conflicts than co-located teams, but also found evidence that shared identify moderated the effect of distribution on task conflict. On the other hand, Bicchieri et al. (2010) found that the type of social interactions mattered for its positive outcome. In a trust game laboratory experiment, they allowed half of their participants to discuss any topic except those pertaining to the game. In the remaining sessions, participants were constrained to only talk about topics identified by the researchers, and that were not relevant to the game. Their results showed that the type of communication (i.e., whether the type of communication was restricted or unrestricted), and not the medium through which participants communicated, had a significant impact on first-mover investments (they made significantly higher investments in the unrestricted conditions).

Even if it seems implicitly assumed in most of the above studies that social interactions will reduce opportunistic behavior and increase cooperative behavior, this has not been the explicit focus of the above studies. However, the theories do suggest that social interactions are essential for how people adopt media, and that positive, social interactions may neutralize the (potentially negative) effects of media. Based on this, we argue:

Hypothesis 3: Employees who experience a social interaction will behave more cooperatively, regardless of the richness of the media through which they interact.

Social interaction theories provide evidence for the importance of developing social interactions. While most hold that social interactions have a generally positive outcome, we also found evidence (e.g., Bicchieri et al., 2010) that the type of social interaction matters. From the literature (e.g., Mortensen and Hinds, 2001; Hinds and Mortensen, 2005) it would seem that trust and strong social identity are essential factors for social interactions to result in a positive outcome. However, the mechanisms that facilitate either trust or social identities or other identifiers of positive social interactions, and not the least, whether these mechanisms are similar for collocated and distributed teams, are not clearly defined.

### Interaction Adaptation and Cooperative vs. Self-interested Behavior

One field of study that holds potentially interesting insights into the mechanisms underlying the positive development of social interaction, but which has not yet been integrated into the study of distributed organizations, is interaction adaptation theory (Burgoon et al., 2007). Interaction adaptation theory focuses on how individuals coordinate their communication behaviors temporally with those of another conversant to achieve a kind of “goodness of fit” between them” (Burgoon et al., 2007, p. 19). As described by Condon and Ogston (1971), this “goodness of fit,” or synchrony, relates to the cyclical nature of behaviors rather than similarities between the behaviors themselves.

One central perspective to the study of interpersonal adaptation is the joint action literature, relating to the study of social interaction whereby interactors coordinate their actions in space and time (Sebanz et al., 2006, p. 70). Numerous joint action studies have demonstrated how people not only have a natural tendency to imitate, mirror, or mimic each other’s physical expressions, but also that people whose motor behaviors are synchronized tend to feel higher affiliation, liking, and generally experience the interaction as smoother (Bernieri, 1988; Hove and Risen, 2009; Lakens and Stel, 2011). Such synchronization, even if people are not consciously aware of it, has been shown to have positive effects on people’s efficiency in joint action tasks (Wiltermuth and Heath, 2009). Synchronization therefore has been argued to capture an innate human ability to adapt to others, an ability which humans have inherited evolutionarily for enhanced efficiency in social interactions (Hove and Risen, 2009). We adapt to others to better understand and to make our behaviors suit those of others for better accomplishment of joint goals.

Studies have shown how individuals through social interaction synchronize not only their motor behaviors, but also their heart rates (Konvalinka et al., 2011; Mitkidis et al., 2015); and their skin conductance responses (Mønster et al., 2015). It has been suggested that synchrony should be perceived as a psychophysiological measure of shared emotions (Konvalinka et al., 2011; Håkonsson et al., 2015; Mønster et al., 2015). Consistent with this, Mønster et al. (2015) found that high synchronization also was predictive of high perceived coherence in teams, just as Mitkidis et al. (2015) found that high degrees of heart-rate synchrony in an unrelated joint task was predictive of high mutual trust, expressed in terms of a public goods game.

As another measure of trust, research has shown that when one is trusted, one’s brain produces oxytocin in proportion to the degree of trust shown (Zak et al., 2005; Morhenn et al., 2008). Zak and Knack (2001) argue that trust affects an organization’s ability to accomplish its objectives because trust acts as an economic lubricant, easing the social interactions necessary to meet goals. Following Zak et al. (2005), the brain chemical oxytocin increases awareness of others’ emotional states. This awareness in turn makes people more effective at cooperating. Further, the amount of oxytocin is a rapid brain signal, turning on when we are shown trust, and shutting down during periods of high stress or extreme competition (Zak, 2012).

The above studies point to the innate human ability to adapt to others, an ability that may even explain the efficiency of social interactions. It is an ability that has evolved evolutionarily, long before different information technologies appeared. This ability seems to provide a potentially relevant understanding of the conditions that promote cooperation as well as the conditions that lead to a breakdown of cooperation. Based on the above, it would seem that interaction adaptation is essential for employees to develop a cooperative mindset. Therefore we hypothesize:

Hypothesis 4: Employees who experience interactional adaptation will behave less opportunistically than employees who do not experience interactional adaptation, regardless of the media through which they interact.

Despite its promising insights, the effects of coherence have not yet been fully explored, and no work has tested whether interactional adaptation may occur across different media. Even if new media such as Skype and TelePresence have altered traditional understandings of technologically mediated communications (see Daft and Lengel, 1986), such media still do not provide the same visual, sensory, and olfactory cues that face-to-face interaction does, and therefore may not enable employees to synchronize in the same way.

## Toward an Integrative Model

**FIGURE 1:**
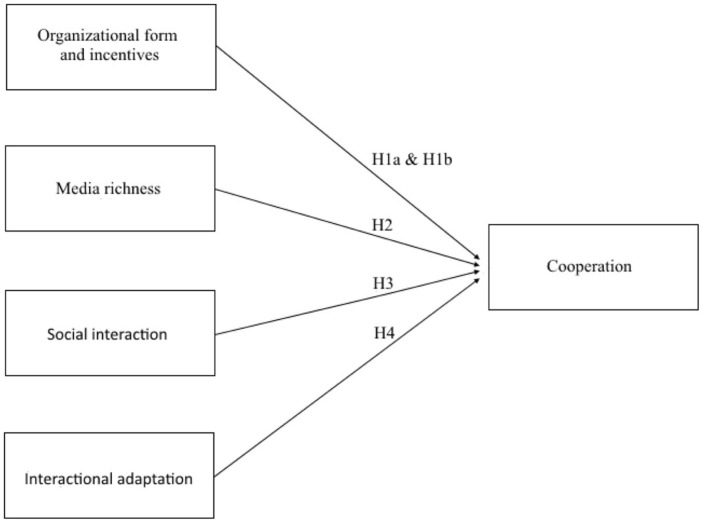
**Main hypotheses from current literature**.

Each of the four theoretical approaches—organization design theory, media richness theory, social interaction, and interaction adaptation theory—provides relevant insights toward an understanding of the conditions that further opportunistic behavior vs. cooperative behavior in distributed decision-making. The four hypotheses developed above are contained in **Table 1**. Their relationships are illustrated in **Figure 1**.

**Table 1:** Main hypotheses.

	Supporting literature	Non-supporting literature
**Main hypotheses**
*Hypothesis 1a:* An incentive system based upon corporate profit induces lower opportunism than an incentive system based upon divisional profit.	Armour and Teece, 1978; Williamson and Ouchi, 1980; Williamson, 1985; Burton and Obel, 1988	
*Hypothesis 1b:* When opportunism is present it will have a greater negative effect on corporate profit in the U-form than in the M-form.	Armour and Teece, 1978; Williamson and Ouchi, 1980; Williamson, 1985; Burton and Obel, 1988	
*Hypothesis 2*: Employees who interact via media of high richness will behave less opportunistically than employees who interact via media of low richness.	Turnbull et al., 1976; Daft and Lengel, 1986; Jarvenpaa and Leidner, 1998; Baltes et al., 2002; Bos et al., 2002; Hinds and Bailey, 2003; Jarvenpaa et al., 2004; Hinds and Mortensen, 2005; Dennis et al., 2008; Naquin et al., 2008; Rockmann and Northcraft, 2008; Hill et al., 2009	Lewis and Fry, 1977; Carnevale et al., 1981; Watson et al., 1988; Swaab and Swaab, 2009
*Hypothesis 3*: Employees who experience a social interaction will behave more cooperatively, regardless of the richness of the media through which they interact.	Barley, 1986, 1988; Watson et al., 1988; Schmitz and Fulk, 1991; Walther, 1992, 1994; Fulk, 1993; DeSanctis and Poole, 1994; Markus, 1994; Orlikowski and Yates, 1994; McGinn and Keros, 2002; Hinds and Mortensen, 2005; Wilson et al., 2006	Bicchieri et al., 2010
*Hypothesis 4:* Employees who experience interactional adaptation will behave less opportunistically than employees who do not experience interactional adaptation, regardless of the media through which they interact.	Zak and Knack, 2001; Zak et al., 2005; Morhenn et al., 2008; Hove and Risen, 2009; Wiltermuth and Heath, 2009; Konvalinka et al., 2011; Zak, 2012; Håkonsson et al., 2015; Mitkidis et al., 2015; Mønster et al., 2015	Wiltermuth, 2012

As also appears from **Table 1** and from **Figure 1**, the different perspectives have not yet been integrated into a comprehensive understanding of cooperative vs. self-interested behavior in distributed teams. Even if all of the theories reviewed contain important insights, none of the four approaches fully accounts for all potentially influential aspects. This does not mean that the theories are not valid; indeed, they have all been empirically demonstrated to be relevant.

The organizational design literature seems clear—organizational form and incentives matter, and it is possible to reduce opportunistic behavior through organizational form and incentives. Yet, the studies have not been extended to virtual contexts, so it is not clear whether organizational form and incentives matter to the same extent when people interact via different communication media.

Evidence from the media richness literature indicates that media richness influences whether people behave cooperatively. Even if the media richness theory has not dealt specifically with opportunistic behavior, there is empirical evidence that high media richness promotes cooperation. The media richness theory, however, has been criticized for placing too much emphasis on the type of media used.

Despite the empirical evidence of the media richness approach, the social interaction literature has provided empirical evidence that the richness of the media is less influential. Rather, the social context and social connections are essential to understand people’s use of media. Yet, there are studies (Hancock et al., 2010) that have shown that the type of social interaction is essential for its positive outcomes. This raises the issue of what a “social connection” is—how does it emerge, and what are the conditions for its development?

The interaction adaptation literature shows that people have an innate human ability to adapt to each other, and that when they do, they produce specific hormones or develop physiological synchrony. Whether or not such interactional adaptation occurs might be predictive of the positive effects of the social interactions. But the interaction adaptation literature has, to our knowledge, only examined people in face-to-face interactions. This raises the issue as to whether an ability that human beings developed evolutionarily also applies when people interact via communication media of different degrees of richness.

**FIGURE 2:**
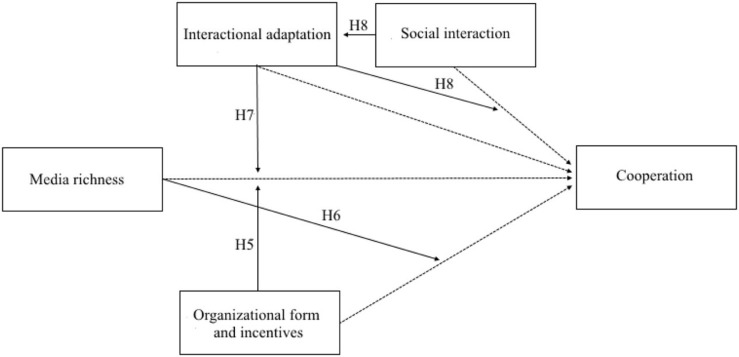
**Main and interactional hypotheses**.

Overall, it seems that the theories are complementary rather than contradictory. While it is not possible to determine from current literature how the theories interact, **Figure 2** illustrates an overview of possible interactional relationships between the main constructs, derived by the theories reviewed.

**Figure 2** acknowledges the literature’s established relationships: that media exerts an influence on employees’ cooperative behavior; that organizational form and incentives reduces opportunism (assumed to increase cooperative behavior); that social interaction often increases cooperation, and that interaction adaptation has been demonstrated to influence cooperation. These relationships are all illustrated by dotted lines in **Figure 2**. Thereby, **Figure 2** contains all the relationships contained in **Figure 1**.

In addition to these established relationships, **Figure 2** also points to additional possible interactions between the constructs that can be derived from the literature, some of which may even explain some of the non-supporting literature findings. These possible interactions are marked by full lines in **Figure 2** and are discussed in the below.

### Interactional Relationships Related to Media Richness, Organizational Form and Incentives, and Cooperation

In the lower part of **Figure 2**, we suggest that the organizational form and incentives to cooperate (Burton and Obel, 1988) will be influenced by the effects of different degrees of media richness. In other words, we expect that organizational form and incentives will moderate the effects of communication media on cooperation (see **Figure 2**). Previous literature has argued that a “cooperative mindset” will moderate the effects of media on cooperation (see Swaab et al., 2012). Here, we argue that organizationally induced incentives to cooperate will have the same effect as “cooperative mindsets.” There is evidence in the literature (De Dreu et al., 2000) that decision makers with a high concern for their own and others’ welfare share more information about underlying interests and priorities than those with egoistic orientations (see also Swaab et al., 2012). Similarly, De Dreu and Carnevale (2003), based on a literature review, concluded that people with a cooperative mindset are more likely to share information. De Dreu and Carnevale (2003) also concluded that decision makers with a cooperative mindset were more prone to interpret others’ actions as efforts to coordinate. This, in turn was found to increase the quality of their interaction outcomes (De Dreu and Carnevale, 2003). Therefore, the literature does not indicate that employees with an incentive to cooperate will be more or less dependent on the presence or absence of visual channels. This can be formulated as:

Hypothesis 5: Employees with an incentive to cooperate will be equally cooperative when interacting via media of high richness or via media of low richness.

On the other hand, there is also literature (e.g., Swaab et al., 2012) indicating that media richness may reinforce incentives not to cooperate. Employees with an incentive not to cooperate are likely to also expect others not to cooperate. When employees with an incentive not to cooperate interact via media of high richness, this may lead them to interpret others’ signals as signs of non-cooperation. Consistent with this, decision makers with non-cooperative orientations have been found more likely to apply competitive tactics when they can see each other compared to when they cannot see each other, and have been found to receive lower joint gain as a consequence of this behavior. For instance, Lewis and Fry (1977), in a laboratory experiment, asked pairs of 46 participants to take the role of buyer and seller to agree on prices for three commodities. They found that a barrier which eliminated visual contact facilitated subjects’ discovery of mutually advantageous alternatives for participants instructed with an individualistic orientation, and had no effect on subjects instructed to consider their opponent’s position (a problem-solving orientation). Similarly, Carnevale et al. (1981), in an experiment involving a similar task to that of Lewis and Fry (1977; i.e., where pairs of 132 participants had to take the role of buyers and sellers and agree on prices), examined the effects of accountability and visual access in integrative bargaining, and found that visual access served as a moderator of the effects of accountability. That is, accountability produced lower joint benefits when negotiators were face-to-face. Cooperative behavior and reports of cooperative atmosphere were also lower when negotiators were face-to-face rather than talking across a barrier. Swaab and Swaab (2009) found that male (but not female) negotiators perceived the presence of eye contact in negotiations as more competitive and threatening and, as a result, shared more information in the absence of eye contact. These results suggest that media of high richness may serve to intensify non-cooperative strategies. In other words, and as depicted in **Figure 2**, this would predict that media richness moderates the relationship between incentives and cooperation.

While the opposite has also been found to be the case—that highly motivated liars interacting in a text-based, computer-mediated environment were more successful in deceiving their partners compared to motivated liars interacting face-to-face (Hancock et al., 2010)—there seems to be convincing evidence supporting the argument that media richness reinforces non-cooperative incentives. Media of high richness convey feelings, not just factual information; therefore their presence may escalate the existence of non-cooperative incentives, in turn leading to less information sharing, and in turn creating a mutually reinforcing strategy of opportunistic behavior. This suggests that:

Hypothesis 6: Decision makers with an incentive to behave opportunistically will behave more opportunistically when interacting via media of high richness than via media of low richness.

### Interactional Relationships Related to Media, Synchrony, Social Connection, and Cooperation

As appears from the upper half of **Figure 2**, another possible relationship is that media richness will influence whether or not employees develop interactional adaptation. Since interaction adaptation is an ability that people have developed evolutionarily, before the emergence of different communication media, it would be interesting to examine whether such adaptation, or synchrony, can emerge when people are not face-to-face, and therefore do not receive the same visual, olfactory, and other stimuli. For instance, it may be that people can only synchronize when they are face-to-face—or when they interact via advanced media such as TelePresence, or perhaps Skype. With these media, employees communicate synchronously. Even if they do not receive olfactory stimuli, they still have eye contact. Such examinations might even lead to an extension of the media richness theory (Daft and Lengel, 1986).

Synchrony is a measure of social connection, or shared emotions (Konvalinka et al., 2011; Mønster et al., 2015), and of trust (Mitkidis et al., 2015). If synchrony only occurs when people interact via media of high richness, this may also provide evidence to previous findings related to the importance of prior acquaintance (Oshri et al., 2008; Hinds and Cramton, 2013). As these studies have demonstrated, having once met in person reduces subsequent problems related to communication via media. Synchrony might be one mechanism that drives this—employees who once have developed synchrony may experience a cohesive bonding that makes it easier to trust each other later on. Hence, one possible hypothesis is that:

Hypothesis 7: Employees who interact via media of high richness will have an easier time developing synchrony than employees who interact via media of low richness.

It may also be possible to relate interactor adaptation to the lack of consistence in studies related to social interactions and cooperation, as interactor adaptation might be an explanatory factor for whether or not social interactions lead to cooperative behaviors, regardless of the media. While there is not existing literature that examines these matters, one might expect that if there is interactor adaptation, employees will cooperate. If there is not, they will not cooperate. This would suggest a mediating effect on the relationship between media richness and cooperation:

Hypothesis 8: The effect of social interactions on cooperation is mediated by the degree of synchrony between employees.

The above interactional hypotheses are included in **Table 2**, together with the main hypothesis derived in the first part of the paper. In **Table 2**, Hypotheses 6–8 constitute the possible interactional relationships that we illustrated in **Figure 2**, and that were discussed in the above.

**Table 2:** Main and interaction hypotheses.

	Supporting literature	Non-supporting literature
**Main hypotheses**
*Hypothesis 1a:* An incentive system based upon corporate profit induces lower opportunism than an incentive system based upon divisional profit.	Armour and Teece, 1978; Williamson and Ouchi, 1980; Williamson, 1985; Burton and Obel, 1988	
*Hypothesis 1b:* When opportunism is present it will have a greater negative effect on corporate profit in the U-form than in the M-form.	Armour and Teece, 1978; Williamson and Ouchi, 1980; Williamson, 1985; Burton and Obel, 1988	
*Hypothesis 2*: Employees who interact via media of high richness will behave less opportunistically than employees who interact via media of low richness.	Turnbull et al., 1976; Daft and Lengel, 1986; Jarvenpaa and Leidner, 1998; Baltes et al., 2002; Bos et al., 2002; Hinds and Bailey, 2003; Jarvenpaa et al., 2004; Hinds and Mortensen, 2005; Dennis et al., 2008; Naquin et al., 2008; Rockmann and Northcraft, 2008; Hill et al., 2009	Lewis and Fry, 1977; Carnevale et al., 1981; Watson et al., 1988; Swaab and Swaab, 2009
*Hypothesis 3*: Employees who experience a social interaction will behave more cooperatively, regardless of the richness of the media through which they interact.	Barley, 1986, 1988; Watson et al., 1988; Schmitz and Fulk, 1991; Walther, 1992, 1994; Fulk, 1993; DeSanctis and Poole, 1994; Markus, 1994; Orlikowski and Yates, 1994; McGinn and Keros, 2002; Hinds and Mortensen, 2005; Wilson et al., 2006	Bicchieri et al., 2010
*Hypothesis 4:* Employees who experience interactional adaptation will behave less opportunistically than employees who do not experience interactional adaptation, regardless of the media through which they interact.	Zak and Knack, 2001; Zak et al., 2005; Morhenn et al., 2008; Hove and Risen, 2009; Wiltermuth and Heath, 2009; Konvalinka et al., 2011; Zak, 2012; Håkonsson et al., 2015; Mitkidis et al., 2015; Mønster et al., 2015	Wiltermuth, 2012
**Possible interaction hypotheses**
*Hypothesis 5:* Employees with an incentive to cooperate will be equally cooperative when interacting via media of high richness or via media of low richness.	Burton and Obel, 1988; De Dreu et al., 2000; De Dreu and Carnevale, 2003; Swaab et al., 2012	
*Hypothesis 6:* Decision makers with an incentive to behave opportunistically will behave more opportunistically when interacting via media of high richness than via media of low richness.	Lewis and Fry, 1977; Carnevale et al., 1981; Swaab and Swaab, 2009; Swaab et al., 2012	Hancock et al., 2010
*Hypothesis 7:* Employees who interact via media of high richness will have an easier time developing synchrony than employees who interact via media of low richness.	Daft and Lengel, 1986; Oshri et al., 2008; Konvalinka et al., 2011; Zak, 2012; Hinds and Cramton, 2013; Mitkidis et al., 2015; Mønster et al., 2015	
*Hypothesis 8:* The effect of media on cooperation is mediated by the degree of synchrony between employees.		

It does seem that Hypotheses 6–8 are able to explain some of the literature’s previously contradictory findings. Hypothesis 5 predicts that incentives and organizational form will overrule the effects of media richness such that media, under such conditions, will not have its predicted effects. Similarly, Hypotheses 7 and 8 predict that social interactions might overrule the effects of media on cooperative behavior. Moreover, Hypothesis 8 proposes that the reason why social interactions may not always lead to cooperative behavior can be explained by whether or not the social interactions are such that they lead to interaction adaptation. While there is support for these arguments in the literature, this is of course subject to be demonstrated, and, it would seem, a subject worthy of future studies.

## Conclusion

We examined four major streams of literature with relevance for explaining self-interest vs. cooperation in distributed organizations, i.e., in organizations where communication is achieved via media of different degrees of richness.

From the review, it is apparent that all the lines of evidence contain relevant insights. At the same time, some of the literature also held contradictory findings. Finally, no literature, to our knowledge, had examined the joint effects of the four different perspectives.

Organizational design studies hold that incentives may further cooperative behavior, but their applicability has not been systematically examined across different communication media. Media richness theory has been criticized for being deterministic, and for not incorporating evidence that a positive connection, or a shared group identity, can overcome the difficulties that employees sometimes face in the absence of communication channels. Accordingly, social interaction theories contradict the richness approach by showing that the absence of visual, vocal, and synchronous communication channels does not necessarily deteriorate social interactions. Social interaction theories, however, are not very specific in terms of identifying whether social interactions always have positive outcomes. Here, interaction adaptation theories would seem to hold potentially relevant insights, as they maintain that it is the interactors’ adaptation, measurable by trust, synchronization, or hormonal levels that matters, not necessarily the social interaction in itself.

Overall, therefore, our review of the current literature led us to conclude that none of the theories is sufficient in itself, and that the theories should be reconciled for a more complete understanding of cooperation vs. opportunism and their antecedents.

Toward this purpose, our paper suggested a set of possible interaction hypotheses that all had support from the literature, and that also might explain some of the previous findings’ contradictory results. Whether these hypotheses hold evidence is naturally the subject of future empirical studies.

Given that self-interest vs. cooperation is a fundamental dilemma in organizational behavior, and given the fact that more and more firms communicate via different media, some of which hold technological properties that are substantially different from when the media richness theory was conceived in the 1980’s, does seem a worthy subject of future studies.

## Author Contributions

All authors listed, have made substantial, direct and intellectual contribution to the work, and approved it for publication.

## Conflict of Interest Statement

The authors declare that the research was conducted in the absence of any commercial or financial relationships that could be construed as a potential conflict of interest.

## Footnotes

1 http://dictionary.reference.com

2 Of course, these arguments are based on the likeliness of a given media to breed opportunism, and not reflecting the way the availability of, for example, e-mail or conferencing makes it possible to interact with people with whom one would otherwise not interact at all. Nevertheless, even if the very availability of media may decrease opportunism overall, our hypothesis relates to the relative likeliness of opportunistic behavior across different media.
